# EDIFY (Eating Disorders: Delineating Illness and Recovery Trajectories to Inform Personalised Prevention and Early Intervention in Young People): project outline

**DOI:** 10.1192/bjb.2022.83

**Published:** 2023-12

**Authors:** Amelia Hemmings, Helen Sharpe, Karina Allen, Heike Bartel, Iain C. Campbell, Sylvane Desrivières, Richard J.B. Dobson, Amos A. Folarin, Tara French, Jonathan Kelly, Nadia Micali, Sneha Raman, Janet Treasure, Ruby Abbas, Beck Heslop, Tallulah Street, Ulrike Schmidt

**Affiliations:** 1 Institute of Psychiatry, Psychology and Neuroscience, King's College London, London, UK; 2 University of Edinburgh, Edinburgh, UK; 3 South London and Maudsley NHS Foundation Trust, London, UK; 4 University of Nottingham, Nottingham, UK; 5 Institute of Health Informatics, University College London, London, UK; 6 Health Data Research UK London, University College London, London, UK; 7 National Institute for Health and Care Research Biomedical Research Centre at South London and Maudsley NHS Foundation Trust and King’s College London, London, UK; 8 National Institute for Health and Care Research Biomedical Research Centre at University College London NHS Foundation Trust, London, UK; 9 Glasgow School of Art, Glasgow, UK; 10 Beat, Norwich, UK; 11 University College London, London, UK; 12 Psychiatric Research Centre Ballerup, Ballerup, Denmark; 13 EDIFY, London, UK

**Keywords:** Eating disorders, interdisciplinary working, prevention and early intervention, youth engagement, risk and resilience

## Abstract

EDIFY (Eating Disorders: Delineating Illness and Recovery Trajectories to Inform Personalised Prevention and Early Intervention in Young People) is an ambitious research project aiming to revolutionise how eating disorders are perceived, prevented and treated. Six integrated workstreams will address key questions, including: What are young people's experiences of eating disorders and recovery? What are the unique and shared risk factors in different groups? What helps or hinders recovery? How do the brain and behaviour change from early- to later-stage illness? How can we intervene earlier, quicker and in a more personalised way? This 4-year project, involving over 1000 participants, integrates arts, design and humanities with advanced neurobiological, psychosocial and bioinformatics approaches. Young people with lived experience of eating disorders are at the heart of EDIFY, serving as advisors and co-producers throughout. Ultimately, this work will expand public and professional perceptions of eating disorders, uplift under-represented voices and stimulate much-needed advances in policy and practice.

In Western countries, one in every six females and one in twenty males develop an eating disorder.^1,2^ Prevalence and burden of eating disorders are rising, with these disorders developing earlier and hospital admissions increasing.^1,3^ Anorexia nervosa has the highest mortality of any mental disorder; 1 in 2–3 people with bulimia nervosa or binge eating disorder are obese or will become obese, with potential metabolic complications.^1^ Psychological comorbidities are common and contribute to adverse outcomes.^1^ Peak onset of eating disorders is in adolescence, a developmentally sensitive time,^1^ giving eating disorders the power to derail physical, cognitive, socioemotional and educational development. Families and partners are also adversely affected. Estimates suggest a disease burden comparable to that of anxiety and depression,^4^ yet a recent report by the All-Party Parliamentary Group on eating disorders found that between 2015 and 2019 only about 1% of UK research funding went to eating disorders.^5^

Eating disorders have environmental and psychobiological risk and maintaining factors. LGBTQIA+, culturally and ethnically diverse young people and those with higher body mass index (BMI) are particularly at risk for eating disorders^6,7^ but less likely to be diagnosed or treated.^8^ An understanding of how sociocultural factors (e.g. food insecurity, racial discrimination) interact with psychobiological factors and comorbidities is lacking. Intervention within about 3 years of eating disorder onset can improve outcomes. Longer illness is associated with cognitive, behavioural and neurobiological changes^9^ that adversely affect illness progression and treatment outcomes.^9,10^ Despite this, the average duration of untreated illness is 2.9 years for anorexia nervosa and 6.5 years for bulimia nervosa and binge eating disorder.^11^

There have been no coordinated efforts at eating disorder prevention and early intervention. Under a quarter of those with eating disorders in the UK receive treatment.^1^ Available interventions are only moderately effective and not tailored to illness stage or individual characteristics. In recognition of this, early intervention for eating disorders is now a UK policy aim.^12^ From cancer to psychosis, early intervention and stage models of illness have been the focus of research, leading to more targeted treatments and better outcomes. Nothing comparable exists in eating disorders. EDIFY aims to address this gap.

## The EDIFY project

EDIFY (Eating Disorders: Delineating Illness and Recovery Trajectories to Inform Personalised Prevention and Early Intervention in Young People) is funded by UK Research and Innovation (UKRI) under their Adolescence, Mental Health and the Developing Mind programme. This programme supports ‘multi and inter-disciplinary research and innovation that address areas of strategic importance aligned with government policy and research priorities’ so as ‘to better understand how and why mental health problems emerge and what makes some young people more susceptible or resilient than others’.^13^ Specifically, it is recommended that creative arts and visual tools are used ‘to both learn from and support young people’. This knowledge will be used to generate evidence that can lead to new approaches for improving adolescent mental health.

EDIFY is a 4-year interdisciplinary programme of research that addresses gaps in knowledge of eating disorder risk and resilience, illness and recovery trajectories, and targets for personalised intervention. By combining multiple perspectives (young people, families, other stakeholders) with cross-disciplinary clinical and academic expertise, we aim to accelerate the depth, quality and speed of knowledge generation in this area.

Six integrated workstreams (WS1–WS6) will address a range of questions, including: WS1: What are young people's experiences of developing an eating disorder, help-seeking and recovery? What are the perspectives of diverse and high-risk groups? WS2: What are the key social and psychobiological risk and resilience factors in eating disorders and how do these interact? What are the unique and shared risk factors across groups? WS3: What helps/hinders recovery in young people with eating disorders? WS4: How do eating disorder behaviours and brain responses change from early- to late-stage illness? WS5: How can we intervene earlier, quicker and in a more personalised way? WS6: How can we co-produce outputs and share our findings with young people, professionals and the public?

In addition to knowledge generation, we anticipate a number of outputs, all co-produced with young people, including: a biopsychosocial model of eating disorder risk/illness stages incorporating evidence of lived experience, illness/recovery trajectories, characteristics of illness stages and mechanisms driving transitions between them; protocols and/or proof of concept of mechanism-focused interventions for young people with different risk profiles/illness stages; and resources and creative outputs to increase public and professional understanding of eating disorders and young people's lived experiences of these conditions. The findings will be used to influence policy in this area.

In this article, we describe our approach to youth involvement and provide an overview of our programme.

## Research culture and values – towards genuine co-production

An innovative aspect of EDIFY, and a core guiding principle, is the involvement of young people throughout, from research design through to knowledge dissemination, ensuring that this work represents the insights and priorities of those most affected by eating disorders.

Advisors with lived experience were involved from EDIFY's inception and made suggestions that shaped the proposal to align with the needs of young people with eating disorders. Throughout the project, each workstream will have three designated young experts by experience, chosen to reflect multiple perspectives and backgrounds. They will work with EDIFY researchers on features such as study design, development of study materials and knowledge mobilisation. They will also form our youth advisory board overseeing the project.

To identify diverse youth advisors, we recruited via national and local eating disorder charities (Beat, First Steps ED), clinical eating disorder services, and Leaders Unlocked, a specialist youth-involvement organisation. To encourage youth engagement, multi-method creative approaches will be used to facilitate experience sharing, and emphasis will be placed on removing hierarchies of power between researchers and young people. Our young people's panel varies in age, education and research familiarity; therefore, we will provide individualised training and support to empower each member. Relevant guidance will be followed to ensure effective co-production with young people and inclusive working with diverse groups.

In addition to remuneration for their time, benefits to young people will include the acquisition of leadership and other transferrable skills (e.g. co-chairing meetings; presentation skills); enhancing their critical ‘literacy’ through debate; and having fun together as part of a community of like-minded young people and within the wider EDIFY group. Young people will also have the opportunity to obtain co-authorship on papers, as well as references for college, university or employers.

In line with the funding objectives, EDIFY foregrounds the voices of young people, but we are mindful of the importance of families/carers in understanding and improving treatments and care. With a range of other stakeholders, families and carers are represented in a second advisory board that interacts with the young people's advisory board.

## An interdisciplinary programme of work

**Fig. 1:**
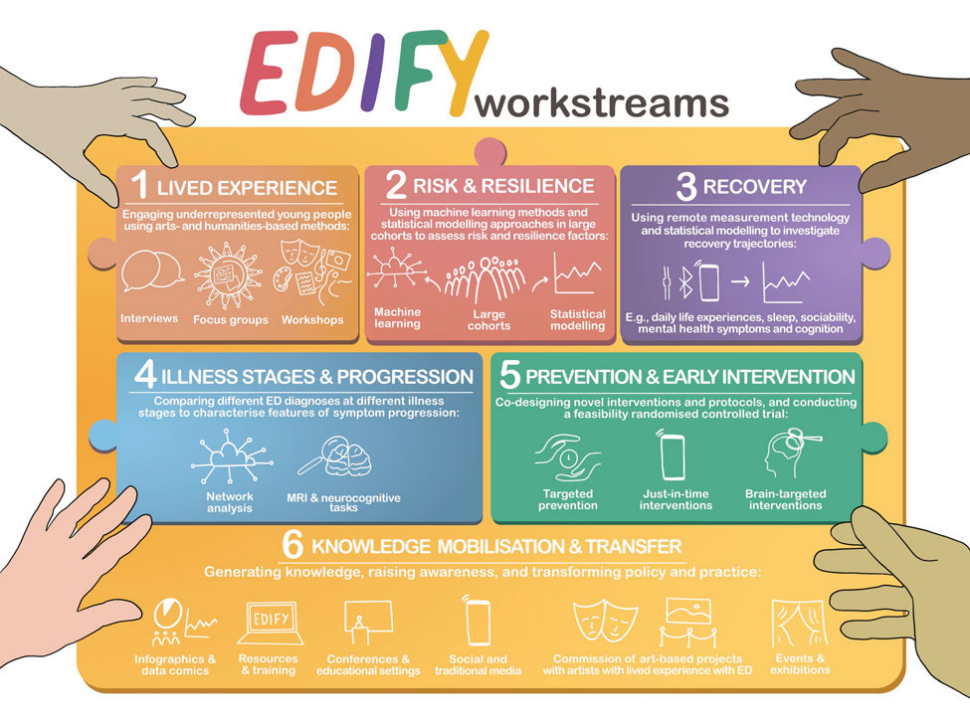
This is a visual depiction of the six EDIFY workstreams (artwork by Dr Mariana Lopes). Each workstream feeds into the others to form a whole picture, to be mobilised in workstream 6, in raising awareness and translating EDIFY findings to real-world policy and practice developments to help young people with eating disorders.

**Fig. 2:**
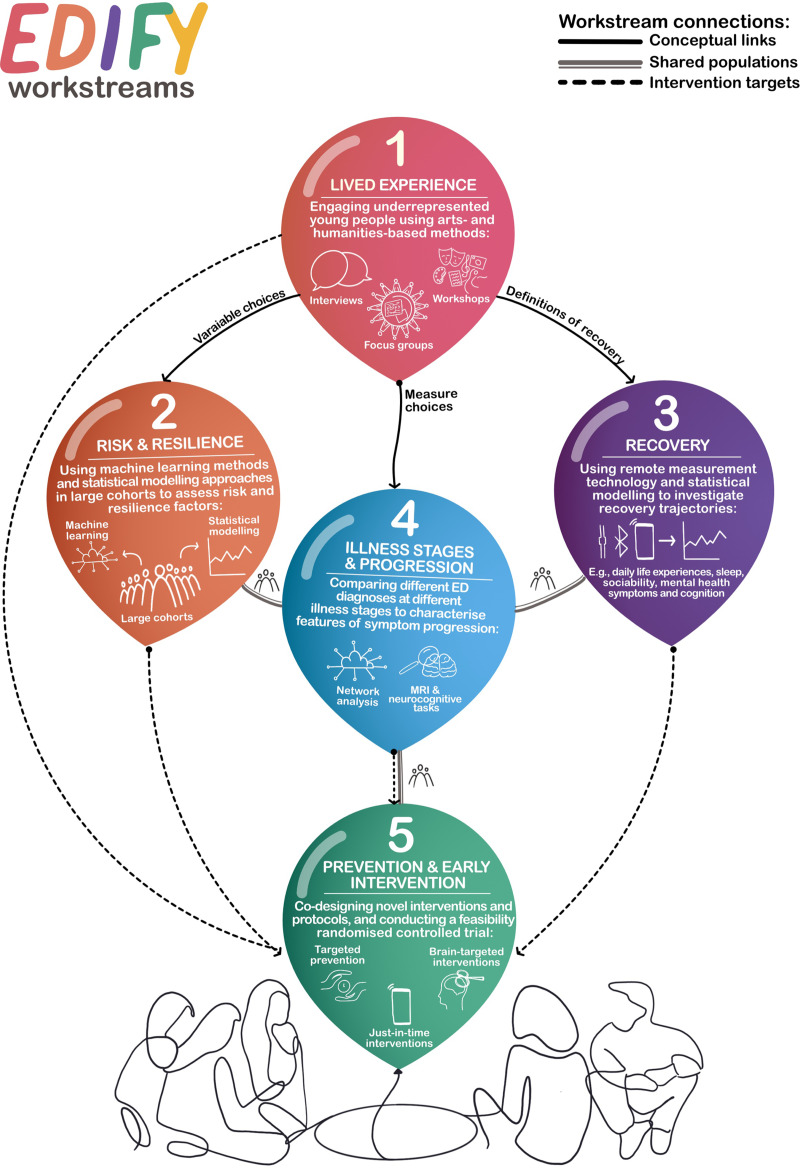
A graphical representation of the five central scientific workstreams within EDIFY (artwork by Dr Mariana Lopes). This shows the methods and techniques of each (in the balloons) and the connections they share. These connections are fluid, to facilitate knowledge-sharing between workstreams and keep the voice of young people with lived experience central. The sixth workstream is not included in this diagram but is dedicated to knowledge mobilisation of findings from workstreams 1–5.

Our programme consists of six workstreams (Figs 1, 2) that will be integrated and co-developed over time. In particular, the arts-, design- and humanities-led workstreams (WS1 and WS6) are cross-cutting, feeding into and drawing from, the other workstreams throughout the programme. To facilitate integration and learning, we will conduct scoping, systematic or meta-analytic reviews on programme-wide topics (e.g. eating disorders and developmental factors). In addition to adhering to common measurements for mental health, we will agree core measures across workstreams and replicate existing protocols to allow cross-validation and harmonisation of data-sets within and outside EDIFY. To maximise value, impact and integration, workstreams have multiple points of synergy, including shared participants, shared assessments and common conceptual foci, such as vulnerability to eating disorders, recovery and illness stage.

### WS1: Lived experience

This cross-cutting workstream aims to shed light on young people's experiences of help-seeking, treatment, transitions and recovery, with a focus on diverse, under-represented and high-risk groups. This will include young people from diverse cultural and ethnic backgrounds, LGBTQIA+ individuals, males, those with higher BMI, young people not in education, employment or training (NEETs) and those from low socioeconomic and rural backgrounds. As young people may occupy multiple marginalised social identities, an intersectional approach will be taken.^14^

**Fig. 3:**
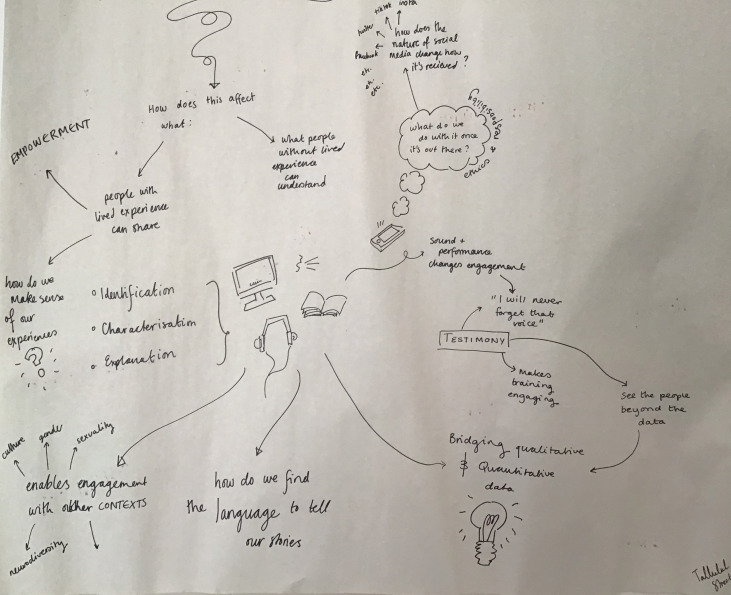
Visual notes taken at a workstream 1 meeting by one of the authors (T.S.).

Qualitative, design-led and arts-based methodologies will be employed to co-create narrative accounts of illness and recovery with young people. Data will be collected via interviews, focus groups and workshops, as well as artistic outputs and existing sources such as social media posts, stories and music. Creative engagement throughout the process will allow participants to ‘build’ and share their experience such that it is meaningful to them, enabling visualisation of their eating disorder journeys, identifying enablers and barriers to help-seeking. This will allow collaboration and identification of shared illness and recovery features. For example, during our first WS1 meeting, a youth advisor took visual notes, a unique combination of text and imagery taken in real time on paper, which allows a more fluent connection between points raised than the more rigid structure of writing (Fig. 3). Harnessing youth advisors’ knowledge, skills and perspective, here by asking them to identify, order and creatively convey key points of the discussion for the whole group, is one example of our commitment to co-production of knowledge with young people and to embracing the new perspectives this presents.

Creative projects undertaken previously by EDIFY investigators in collaboration with, for example, men and boys with eating disorders will further shape our engagement. Examples are Bartel's ‘Hungry for Words’ project,^15^ including video poems, photography, animations and rap music.

Qualitative analysis will be used to synthesise data and convey young people's lived experiences of eating disorders in academic papers.

**Fig. 4:**
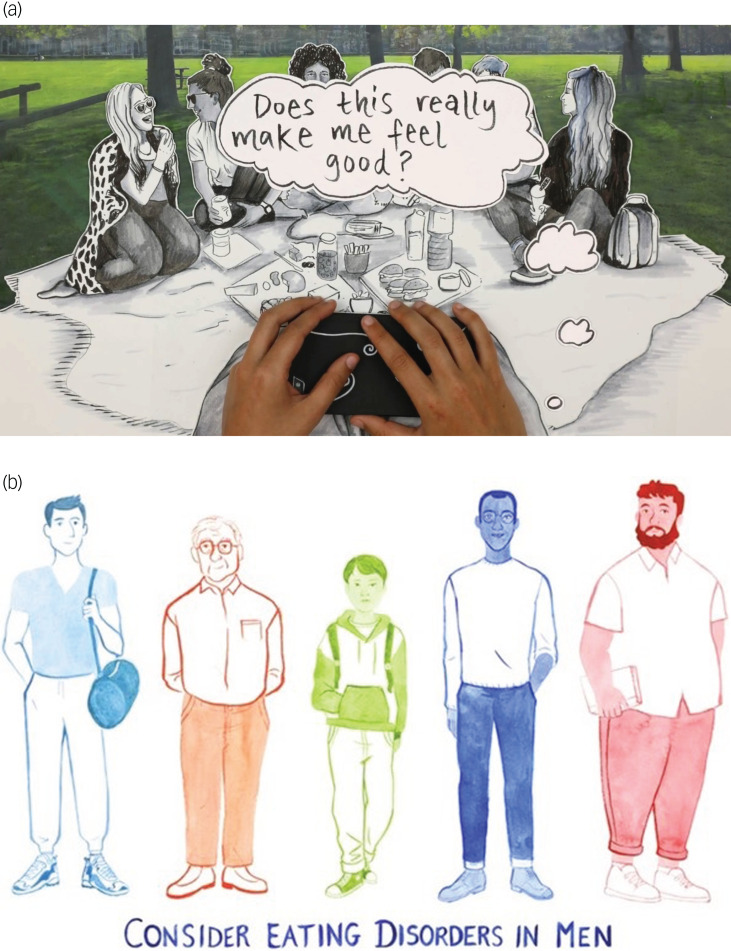
(a) Screenshot from the animation ‘Social Media, Food and Me’, produced by Woven Ink. The full animation can be seen at https://freedfromed.co.uk/news-and-stories/37/social-media-food-and-me. (b) A still from the animation ‘Consider Eating Disorders in Men’, produced by Woven Ink, artwork by Mathilde Laillet. The full animation can be seen on YouTube (search: ‘Consider Eating Disorders in Men’ at www.youtube.com).

Animated films, co-created with young people and Woven Ink (a creative studio, working predominantly with the health and social care sector) will further amplify young people's voices and empower them to tell their stories. Figure 4 shows examples of our previous collaborations with Woven Ink.

WS1 has a reciprocal relationship with all other workstreams, in that it feeds into and informs them, as well as being responsive to learning from them, allowing the voices of young people to shape clinical research.

### WS2: Risk and resilience

Research has documented increasing rates of appearance concerns, dieting and eating disorders in young people in the UK, with COVID-19 restrictions and increased reliance on social media likely exacerbating this.^16^ Although some young people with high-risk behaviours such as dieting will develop an eating disorder, not all risk-exposed youth will. Little is known about which factors trigger eating disorder onset, which may protect against it and whether risk processes differ in under-represented groups.

WS2 will use a minimum of seven community (IMAGEN (imagen-project.org); EDGI (edgiuk.org); the Avon Longitudinal Study of Parents and Children (ALSPAC); the British Household Panel Survey (BHPS); the Millennium Cohort Study) or clinical (ESTRA;^17^ STRATIFY) cohorts to develop and test predictive models of risk and resilience for eating disorders and associated behaviours in young people. Alongside existing research, this workstream will identify key areas of priority based on findings from WS1 (i.e. what young people affected by eating disorders report as being central to their development of eating difficulties). We will use several modelling approaches, including machine learning methods, to determine biopsychosocial markers of eating disorders and disentangle predictors from correlates of illness. We will explore whether the same factors predict onset of high-risk behaviours (e.g. appearance concerns, dieting) versus progression from high-risk behaviours to eating disorders, as well as how young people's membership of various or multiple marginalised groups influences these models of risk.

Use of multiple, complementary cohorts offers the opportunity to explore an array of potential predictors, spanning biological, psychological and social factors. Models can be cross-validated between general population and clinical samples, and the shared and unique risk factors across different contexts can be unpicked.

### WS3: Recovery

Recovery from an eating disorder is often conceptualised as a process, but details are poorly understood. More understanding of the recovery process is needed to tailor interventions to individuals’ needs. Remote measurement technology (RMT) uses sensors within smartphones and wearable devices to unobtrusively measure human behaviour and physiology (passive RMT) or actively measure daily experiences (active RMT). RMT can help elucidate the recovery process by providing real-time multidimensional information about patients’ clinical state, as well as potentially predicting recovery or relapse. RMT has been used to investigate the trajectories of other psychiatric illnesses,^18^ and a review into its use in eating disorder populations has suggested that it should be used in more naturalistic, everyday settings and for those with bulimic-type eating disorders.^19^

We will prospectively recruit a cohort of 600 young people: 480 with anorexia nervosa or bulimic-type eating disorder (bulimia nervosa, binge eating disorder) from the network of FREED early intervention services in England, and 120 healthy young people via social media and community organisations. Participants will be provided with activity monitors and android smartphones; data will be collected via the RADAR-base platform (radar-base.org).

Passive RMT will be assessed continually, gathering a range of physiological and social data. Active RMT will be collected every 2 weeks (eating disorder symptom, psychological and cognitive measures). An experience sampling methodology (ESM) schedule will be delivered every 6 weeks to provide a more detailed understanding of eating disorder symptom and mood changes and daily stressors. Measures of psychological and social impairment, as well as genetic, neuroimaging and cognitive measures, will be taken. Outcome measures will be completed at baseline and at 3, 6 and 12 months. We will also evaluate user experience through qualitative interviews with selected young people.

### WS4: Illness stages and progression

Neuroimaging, neurocognitive and behavioural studies indicate that eating disorders change as the illness progresses, becoming increasingly neurocognitively ingrained, and that treatment response may become muted over time.^9,10^ For example, research examining illness progression in anorexia nervosa has shown that food choices become more dysfunctional.^20^ General neurocognitive impairments also intensify in those with longer duration of illness.^21^ However, these studies tend to compare adolescents with adults, i.e. they are confounded by developmental stage. Less is known about eating disorders other than anorexia nervosa, which we will attempt to remedy.

This workstream will investigate behavioural and neural differences between early- and later-stage eating disorders, and which factors predict outcomes. We will use data from the ESTRA cohort, as well as recruiting new participants, both healthy individuals and those with early- or late-stage eating disorders, including anorexia nervosa and bulimia nervosa. This will allow comparison of biopsychosocial and cognitive profiles of young people with early- and late-stage eating disorders, to examine changes over time, within and across eating disorder diagnoses.

A variety of demographic, personality, eating disorder psychopathology, cognitive and neuroimaging data will be collected. Statistical modelling techniques will be employed to characterise eating disorder symptoms during illness progression and identify predictors of outcome. We will use network analysis to conceptualise factors and their associations to represent eating disorder psychopathology. To gain mechanistic insights and reveal differences that characterise eating disorder subgroups and illness progression, analyses will include multiple comparisons: between healthy controls and eating disorder subgroups, between patient groups with different illness durations, and between initial and follow-up assessments.

### WS5: Prevention and early intervention

A large evidence base supports the use of universal, targeted and indicated preventive psychological interventions in young people, such as reducing body dissatisfaction.^22^ However, neurobiological risk factors (e.g. neurocognitive markers of eating disorders) may also present an opportunity for illness prevention, in eating disorders or transdiagnostically. Findings from WS2 and WS4 will be used to co-design at least two novel intervention protocols for different illness or developmental stages, with the potential to inform targeted, indicated and/or tertiary prevention of eating disorders. Findings from WS3 will also inform development of a just-in-time adaptive intervention that can be delivered via smartphone. Finally, a road map of promising intervention targets will be created to catalyse future studies/work.

Limited efficacy of current eating disorder treatments^1^ indicates the need for tertiary prevention research, i.e. interventions aiming to facilitate recovery or reduce deterioration when first-line approaches have been unsuccessful. To develop innovative treatments for young people with persistent eating disorders, we will explore the use of non-invasive neuromodulation in this group. This will be based on our studies in adults with persistent anorexia nervosa where repetitive transcranial magnetic stimulation (rTMS) has promising clinical outcomes, with substantial improvements in mood, quality of life, eating disorder symptoms and BMI.^23^ This work also provided insight into likely mechanisms of action, namely, rTMS normalised gaze avoidance of food stimuli, lessened excessive self-regulatory control over food choices,^20^ and there was a significant decrease in amygdala cerebral blood flow in the real rTMS group which correlated with longer-term weight gain.^24^ The intervention was highly acceptable to patients.^25^

We will use intermittent theta-burst stimulation (iTBS): it is widely used for treating depression, produces similar effects to rTMS, is better tolerated and takes less time to administer. Uptake, retention and acceptability will be assessed, and treatment effect sizes for mood and BMI will be obtained, informing a larger future trial. We propose that iTBS promotes neuroplasticity and changes the relationship between the dorsolateral prefrontal cortex (DLPFC) and amygdala. DLPFC stimulation may reduce negative emotional responses to food cues in anorexia nervosa via this mechanism, as suggested by the findings of our adult trial.^20^

### WS6: Knowledge mobilisation and transfer

In collaboration with young people and partner organisations, this cross-cutting workstream will focus on producing a range of messages and activities tailored to different audiences, to raise awareness and stimulate change in policy and practice. These will include infographics, data comics and an EDIFY website (edifyresearch.co.uk) with resources for both public and professional audiences, as well as training for professionals. Findings will be disseminated in conferences, educational settings and via social and traditional media. Impact will be evaluated in terms of reach, usefulness, use and partnership indicators. Led by EDIFY findings and in partnership with young people, we will commission three arts-based projects aimed at raising awareness and spreading knowledge. Links with different artists with lived experience of eating disorders will facilitate diverse projects across different regions. Events and exhibitions will be held at the Bethlem Museum of the Mind (Beckenham), the Science Gallery London and the Glasgow School of Art.

## Conclusions

To produce effective change, all aspects of eating disorders must be investigated and tackled cohesively. This involves a rapprochement and interchange between arts and humanities on the one hand and psychiatry, psychology and neuroscience on the other.^26^ Through adopting such a transdisciplinary, holistic approach, EDIFY aims to transform eating disorder detection, prevention, treatment and services, with the potential for step change in policy and practice for young people with eating disorders. Ultimately, our ambition is that EDIFY's research developments will reshape the public and professional understanding of eating disorders to be more informed, inclusive and hopeful. This is a step towards redressing the lack of parity of esteem^5^ faced by eating disorders and giving those affected by these conditions access to timely, inclusive and evidence-based care.

## Data Availability

Data availability is not applicable to this article as no new data were created or analysed in its preparation.

## About the authors

**Amelia Hemmings** is a research assistant in the Department of Psychological Medicine, Institute of Psychiatry, Psychology and Neuroscience (IoPPN), King's College London (KCL), UK. **Helen Sharpe** is a senior lecturer in applied psychology at the School of Health in Social Science, University of Edinburgh, UK. **Karina Allen** is a consultant clinical psychologist and researcher with the Eating Disorder Service of South London & Maudsley NHS Foundation Trust, London, UK. **Heike Bartel** is Professor of German Studies and Health Humanities in the Faculty of Arts at the University of Nottingham, UK. **Iain C. Campbell** is a Senior Research Fellow in the Department of Psychological Medicine, IoPPN, KCL, UK. **Sylvane Desrivières** is Professor of Biological Psychiatry at the Social, Genetic and Developmental Psychiatry Centre, IoPPN, KCL, UK. **Richard J.B. Dobson** is Professor of Medical Informatics and lead for Bioinformatics and mHealth for the Maudsley Biomedical Research Centre and King's College London, UK. **Amos A. Folarin** is the senior software development group leader at King's College London/National Institute for Health Research Maudsley Biomedical Research Centre, UK. **Tara French** is a visiting Research Fellow at Glasgow School of Art, UK. **Jonathan Kelly** is a policy advisor at Beat, UK. **Nadia Micali** is an honorary senior lecturer at the Great Ormond Street Hospital Institute of Child Health, UCL, and Head of Research at the Mental Health Service of the Capital Region of Denmark, Denmark. **Sneha Raman** is a Research and Teaching Fellow in the Innovation School at Glasgow School of Art, UK. **Janet Treasure** is Professor of Adult Psychiatry in the Department of Psychological Medicine, IoPPN, KCL, UK. **Ruby Abbas** is a member of the EDIFY youth advisory board, working with WS1, London, UK. **Beck Heslop** is a member of the EDIFY youth advisory board, working with WS1, London, UK. **Tallulah Street** is a member of the EDIFY youth advisory board, working with WS1, London, UK. **Ulrike Schmidt** is Professor of Eating Disorders in the Department of Psychological Medicine, IoPPN, KCL, UK.

## References

[1] Treasure J, Duarte TA, Schmidt U. Eating disorders. Lancet 2020; 395: 899–911. 32171414 10.1016/S0140-6736(20)30059-3

[2] Limbers CA, Cohen LA, Gray BA. Eating disorders in adolescent and young adult males: prevalence, diagnosis, and treatment strategies. Adolesc Health Med Ther 2018; 9: 111–6. 30127650 10.2147/AHMT.S147480 PMC6091251

[3] Esposti M D, Ziauddeen H, Bowes L, Reeves A, Chekroud AM, Humphreys DK, et al. Trends in inpatient care for psychiatric disorders in NHS hospitals across England, 1998/99–2019/20: an observational time series analysis. Soc Psychiatry Psychiatr Epidemiol 2021; 57): 1–4. 34951652 10.1007/s00127-021-02215-5 PMC8705084

[4] Paxton SJ, Hay P, Touyz SW, Forbes D, Madden S, Girosi F, et al. Paying the Price: The Economic and Social Impact of Eating Disorders in Australia. Deloitte Access Economics, 2012.

[5] All-Party Parliamentary Group on Eating Disorders. Breaking the Cycle: An Inquiry into Eating Disorder Research Funding in the UK. Beat, 2021 (https://beat.contentfiles.net/media/documents/APPG_Research_Funding_inquiry_report.pdf).

[6] Cheng ZH, Perko VL, Fuller-Marashi L, Gau JM, Stice E. Ethnic differences in eating disorder prevalence, risk factors, and predictive effects of risk factors among young women. Eat Behav 2019; 32: 23–30. 30529736 10.1016/j.eatbeh.2018.11.004 PMC6382562

[7] Diemer EW, Grant JD, Munn-Chernoff MA, Patterson DA, Duncan AE. Gender identity, sexual orientation, and eating-related pathology in a national sample of college students. J Adolesc Health 2015; 57: 144–9. 25937471 10.1016/j.jadohealth.2015.03.003 PMC4545276

[8] Waller G, Schmidt U, Treasure J, Emanuelli F, Alenya J, Crockett J, et al. Ethnic origins of patients attending specialist eating disorders services in a multiethnic urban catchment area in the United Kingdom. Int J Eat Disord 2009; 42: 459–63. 19115370 10.1002/eat.20631

[9] Treasure J, Stein D, Maguire S. Has the time come for a staging model to map the course of eating disorders from high risk to severe enduring illness? An examination of the evidence. Early Interv Psychiatry 2015; 9: 173–84. 25263388 10.1111/eip.12170

[10] Ambwani S, Cardi V, Albano G, Cao L, Crosby RD, Macdonald P, et al. A multicenter audit of outpatient care for adult anorexia nervosa: symptom trajectory, service use, and evidence in support of “early stage” versus “severe and enduring” classification. Int J Eat Disord 2020; 53: 1337–48. 32064663 10.1002/eat.23246

[11] Austin A, Flynn M, Richards K, Hodsoll J, Duarte TA, Robinson P, et al. Duration of untreated eating disorder and relationship to outcomes: a systematic review of the literature. Eur Eat Disord Rev 2021; 29: 329–45. 32578311 10.1002/erv.2745

[12] NHS England. Guidance to Support the Introduction of Access and Waiting Time Standards for Mental Health Services in 2015/16. NHS England, 2016.

[13] UK Research and Innovation. £24 Million Investment into Adolescent Mental Health. UKRI, 2021; 28 Jun (https://www.ukri.org/news/24-million-investment-into-adolescent-mental-health/ [cited 29 Jun 2022]).

[14] Burke NL, Schaefer LM, Hazzard VM, Rodgers RF. Where identities converge: the importance of intersectionality in eating disorders research. Int J Eat Disord 2020; 53: 1605–9. 32856342 10.1002/eat.23371 PMC7722117

[15] University of Nottingham. Hungry for Words: Creative Approaches to Start the Conversation about Eating Disorders in Men. University of Nottingham, 2022. (https://www.nottingham.ac.uk/research/groups/hungry-for-words/index.aspx [cited 29 Jun 2022]).

[16] Robertson M, Duffy F, Newman E, Bravo CP, Ates HH, Sharpe H. Exploring changes in body image, eating and exercise during the COVID-19 lockdown: a UK survey. Appetite 2021; 159: 105062. 33278549 10.1016/j.appet.2020.105062 PMC7711175

[17] King's College London. *ESTRA: Earlier detection and stratification of eating disorders and comorbid mental illnesses*. KCL, 2022 (https://www.kcl.ac.uk/research/estra [cited 22 Jul 2022]).

[18] Melbye S, Kessing LV, Bardram JE, Faurholt-Jepsen M. Smartphone-based self-monitoring, treatment, and automatically generated data in children, adolescents, and young adults with psychiatric disorders: systematic review. JMIR Ment Health 2020; 7(10): e17453. 33118950 10.2196/17453 PMC7661256

[19] Presseller EK, Patarinski AG, Fan SC, Lampe EW, Juarascio AS. Sensor technology in eating disorders research: a systematic review. Int J Eat Disord 2022; 55: 573–624. 35489036 10.1002/eat.23715

[20] Dalton B, Foerde K, Bartholdy S, McClelland J, Kekic M, Grycuk L, et al. The effect of repetitive transcranial magnetic stimulation on food choice-related self-control in patients with severe, enduring anorexia nervosa. Int J Eat Disord 2020; 53: 1326–36. 32309882 10.1002/eat.23267

[21] Shott ME, Filoteo JV, Bhatnagar KA, Peak NJ, Hagman JO, Rockwell R, et al. Cognitive set-shifting in anorexia nervosa. Eur Eat Disord Rev 2012; 20: 343–9. 22492553 10.1002/erv.2172 PMC3755493

[22] Chua JYX, Tam W, Shorey S. Research review: effectiveness of universal eating disorder prevention interventions in improving body image among children: a systematic review and meta-analysis. J Child Psychol Psychiatry 2020; 61: 522–35. 31746023 10.1111/jcpp.13164

[23] Dalton B, Lewis YD, Bartholdy S, Kekic M, McClelland J, Campbell IC, et al. Repetitive transcranial magnetic stimulation treatment in severe, enduring anorexia nervosa: an open longer-term follow-up. Eur Eat Disord Rev 2020; 28: 773–81. 32706502 10.1002/erv.2766

[24] Dalton B, Maloney E, Rennalls SJ, Bartholdy S, Kekic M, McClelland J, et al. A pilot study exploring the effect of repetitive transcranial magnetic stimulation (rTMS) treatment on cerebral blood flow and its relation to clinical outcomes in severe enduring anorexia nervosa. J Eat Disord 2021; 9(1): 84. 34243816 10.1186/s40337-021-00420-w PMC8268186

[25] Dalton B, Austin A, Ching BC, Potterton R, McClelland J, Bartholdy S, et al. ‘My dad was like “it's your brain, what are you doing?”’: participant experiences of repetitive transcranial magnetic stimulation treatment in severe enduring anorexia nervosa. Eur Eat Disord Rev 2022; 30: 237–49. 35150473 10.1002/erv.2890 PMC9304183

[26] Foreman D. Introducing the new culture section of *BJPsych Bulletin*. BJPsych Bull 2021; 45: 1–3. 33504390 10.1192/bjb.2020.128 PMC8058846

